# Calcinosis Cutis Presenting as Multiple Discharging Sinuses in Skin: A Case Report

**DOI:** 10.31729/jnma.9168

**Published:** 2025-07-31

**Authors:** Pralisha Maharjan, Gopi Aryal, Reena Rana, Kricha Pande, Niki Thakur

**Affiliations:** 1 Department of Laboratory Medicine and Pathology, Nepal Mediciti Hospital, Lalitpur, Nepal

**Keywords:** *calcinosis cutis*, *discharging sinuses*, *surgical excision*

## Abstract

Calcinosis cutis is a rare but significant pathological condition that can present diagnostic and therapeutic challenges. It typically presents as firm nodules or plaques with extensive calcifications that lead to ulceration and sinus tract formation. We present a case of 44 years old female with calcinosis cutis presenting as multiple draining sinuses, a rare and severe manifestation of the disease. The patient developed chronic, non-healing ulcers with intermittent discharge of chalky material, significantly impacting quality of life. Diagnosis was confirmed through clinical examination, imaging, and histopathology. Management was challenging, requiring a combination of wound care, medical therapy, and surgical intervention. This case highlights the importance of early recognition and a multidisciplinary approach to prevent complications and improve outcomes in patients with severe calcinosis cutis.

## INTRODUCTION

Calcinosis cutis is a rare condition marked by the deposition of insoluble calcium salts in the skin and subcutaneous tissue due to tissue damage or metabolic derangements. It is classified into five types: dystrophic, metastatic, idiopathic, iatrogenic, and calciphylaxis.^1^ Dystrophic calcinosis, the most common type, occurs in damaged or inflamed tissues with normal calcium and phosphate levels and is often associated with connective tissue diseases like systemic sclerosis and dermatomyositis.^2^ Clinically, it presents as firm, whitish papules, nodules, or plaques that may ulcerate and discharge a chalky material.^3^ Diagnosis relies on clinical assessment, imaging (X-ray, CT scan, or MRI), and histopathological confirmation.^4^ Management focuses on the underlying cause and may include medical therapy with bisphosphonates, calcium chelators, or surgical excision in symptomatic cases.^5^ We report a case of a 44-year-old female with calcinosis cutis presenting as multiple nodules with ulcers and discharging sinuses—an unusual and severe manifestation that underscores diagnostic challenges and highlights key management strategies.

## CASE REPORT

**Figure 1a:**
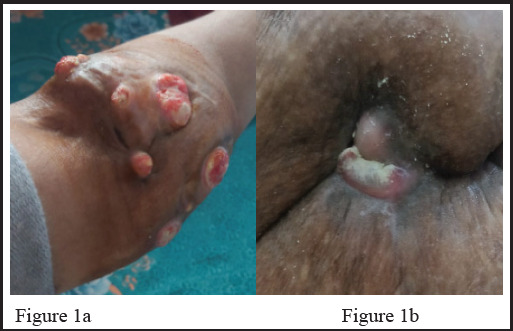
Skin lesion from left elbow showing firm whitish nodules with ulcers along with extruding chalky material. **Figure 1b:** Skin lesion from right base of thigh showing single nodule with ulcer with extruding chalky material.

**Figure 2a:**
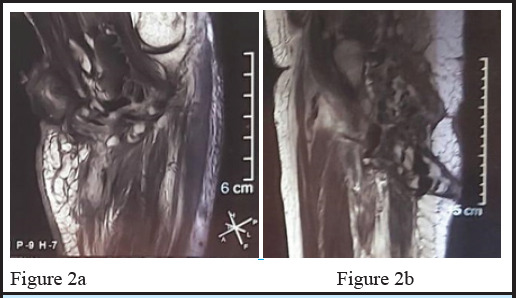
MRI of left elbow showed multiloculated collection with fluid. **Figure 2b:** MRI of right thigh showed showed sinus tract opening in lateral and posterior aspect of thigh, multiloculated collection with fluid.

A 44 years female presented with history of multiple nodules along with chronic, non-healing ulcers and discharging sinuses in right thigh and left elbow for more than 10 years with intermittent discharge of chalky material, significantly impacting her quality of life. Initially the patient had symptoms of nodular lesions in the pressure points starting from right thigh and then in the left elbow which was normal appearing. Gradually the lesion increased in size and skin overlying nodule thinned out, became erythematous and formed ulcer due to continuous friction over the nodule. Later on the ulcers became discharging sinuses in due course of time. On clinical examination, multiple whitish nodular lesion with firm consistency along with ulcers and extruding chalky material varying in size from 1.8×1cm to 4.5cm×3cm were seen in left elbow [Figure 1a]. The lesion in right base of thigh was single, firm white nodule with ulcer along with extruding chalky material measuring 5.5×4.3cm [Figure 1b]. MRI of left elbow showed multiloculated collection and sinus tract in medial aspect elbow, extending to arm and forearm [Figure 2a]. and MRI of right thigh showed sinus tract opening in lateral and posterior aspect of thigh, multiloculated collection with fluid [Figure 2b]. Her hemoglobin was 8gm%,Liver Function Test (LFT), Renal Function Test (RFT) and Thyroid Function Test (TFT) were within normal limit. Serum calcium and serum phosphorus were also normal. Immunological parameters show U1 small nuclear ribonucleoprotein particle (U1-snRNP) and Myositis-specific and associated antibody profile by immunoblot were positive for anti-Mi-2 antibody (++).

**Figure 3a and Figure 3b:**
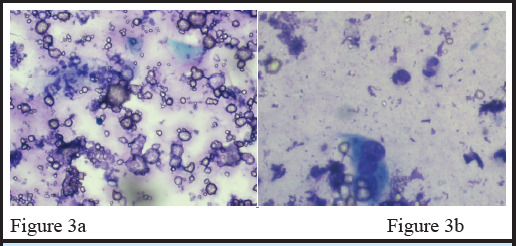
Touch Imprint smear showing calcium crystals along with dense mixed inflammatory infiltrates and multinucleated giant cell in the background. (Giemsa stain x40)

Microscopic examination of touch imprint smear showed dense mixed inflammatory infiltrates along with calcium crystals in the background (Figure 3a and Figure 3b).

**Figure 4a and Figure 4b:**
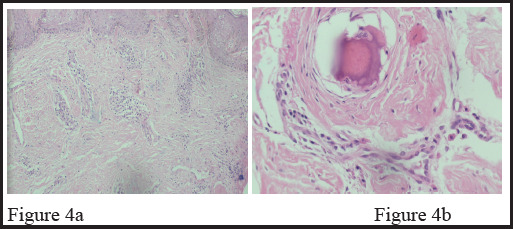
Dermis showed band like infiltrates of plasma cells and lymphoid cells. Deep dermis showed calcified nodules with osseous metaplasia, surrounded by lymphocytes, plasma cells, histiocytes and multinucleated giant cells. (Figure 4a: Hematoxylin and Eosin stain ×10 and Figure 4b: Hematoxylin and Eosin stain ×40).

Microscopic examination of punch biopsy from right hip and left elbow showed skin tissue with dermis and epidermis. Epidermis was unremarkable. Dermis showed band like infiltrates of plasma cells and lymphoid cells. Deep dermis showed calcified nodules with osseous metaplasia, surrounded by lymphocytes, plasma cells, histiocytes and multinucleated giant cells without evidence of granulomas or malignancy (Figure 4a and Figure 4b). Fungal elements or bacterial organism were not identified in PAS and Gram stain.

## DISCUSSION

Calcinosis cutis is a rare but significant condition that can present diagnostic and therapeutic challenges. The pathogenesis involves abnormal calcium deposition in the skin, which may occur due to local tissue damage, systemic metabolic dysregulation, or unknown mechanisms. In our case, the patient exhibited features consistent with dystrophic calcinosis, which is the most common subtype and is often linked to connective tissue diseases such as systemic sclerosis and dermatomyositis. Around 25% to 40% of patients with limited systemic sclerosis develop calcinosis cutis within a decade of disease onset. In dermatomyositis, calcinosis cutis occurs in about 30% of adults and up to 70% of children and adolescents. For patients with systemic lupus erythematosus, periarticular calcifications are observed in 33% of cases, while soft tissue calcifications appear in 17%. ^6^ The diagnostic process relies on clinical examination, imaging, and histopathology. Radiographic imaging, including X-ray, CT and MRI helps identify calcium deposits and their extent. Histological examination confirms the presence of calcium deposits in the dermis and subcutaneous tissue, often surrounded by inflammatory cells. Laboratory investigations are essential to differentiate dystrophic from metastatic calcinosis, as the latter is associated with systemic hypercalcemia and hyperphosphatemia, commonly seen in chronic kidney disease or hyperparathyroidism.^7^ The U1 small nuclear ribonucleoprotein particle (snRNP) is a target of autoreactive B cells and T cells in several autoimmune diseases like rheumatic diseases including systemic lupus erythematosus (SLE) and mixed connective tissue disease (MCTD).^8^ Anti-Mi-2 antibody is typically not linked to calcinosis but rather to skin lesions of dermatomyositis such as Gottron’s sign and Gottron’s papules.^9^ But our case tested positive for both U1snRNP and anti-Mi-2 antibodies along with development of skin nodules, ulcer and discharging sinuses which are rare but severe manifestations of dystrophic calcinosis. Other laboratory investigations like serum calcium, phosphorus, RFT,LFT and TFT were within normal limits which further supported our diagnosis. The pathological reports of previous biopsies reported the case as tumoral calcinosis cutis however after correlating clinical manifestations with radiological and all her laboratory parameters along with histopathological findings we finally made the diagnosis of dystrophic calcification as it is associated with diseases that lead to connective tissue damage which are the major clinical presentation of our patient. Treatment of calcinosis cutis remains challenging and often requires a multimodal approach. Pharmacological interventions include calcium chelators, bisphosphonates, warfarin, and sodium thiosulfate, which have shown varying degrees of success.^10^ In refractory or symptomatic cases, surgical excision of large calcified deposits may be necessary to improve functional outcomes and alleviate pain.^11^ In our patient, conservative management with bisphos-phonates and anti-inflammatory agents was initiated along with surgical excision of nodules in right hip leading to partial resolution of lesions. However, the post treatment status of the patient could not be documented as she was lost to follow-up. Emerging treatments, such as intravenous sodium thiosulfate and biologic agents targeting inflammatory pathways, are being explored for severe or treatment-resistant cases.^12^ However, further clinical studies are needed to establish standardized treatment protocols.
